# Weighing the role of skeletal muscle mass and muscle density in cancer patients receiving PD-1/PD-L1 checkpoint inhibitors: a multicenter real-life study

**DOI:** 10.1038/s41598-020-58498-2

**Published:** 2020-01-29

**Authors:** Alessio Cortellini, Federico Bozzetti, Pierpaolo Palumbo, Davide Brocco, Pietro Di Marino, Nicola Tinari, Michele De Tursi, Veronica Agostinelli, Leonardo Patruno, Cristina Valdesi, Manuela Mereu, Lucilla Verna, Paola Lanfiuti Baldi, Olga Venditti, Katia Cannita, Carlo Masciocchi, Antonio Barile, Jennifer Leigh McQuade, Corrado Ficorella, Giampiero Porzio

**Affiliations:** 1 Medical Oncology, San Salvatore Hospital, University of L’Aquila, L’Aquila, Italy; 2 Department of Biotechnology and Applied Clinical Sciences, University of L’Aquila, L’Aquila, Italy; 3 Faculty of Medicine, University of Milan, Milan, Italy; 4 Radiology Department, St. Salvatore Hospital, L’Aquila, Italy; 5 Clinical Oncology Unit, S.S. Annunziata Hospital, Chieti, Italy; 6 Department of Medical, Oral and Biotechnological Sciences, University G. D’Annunzio, Chieti, Italy; 7 Department of neuroscience, Imaging and clinical Science, University G.D’Annunzio, Chieti, Italy; 8 Section of Integrated Imaging and Radiological Therapies, Department of Neuroscience, University of Chieti, Chieti, Italy; 9 Department of Melanoma Medical Oncology, The University of Texas MD Anderson Cancer Center, Houston, TX USA

**Keywords:** Cancer microenvironment, Tumour immunology

## Abstract

Sarcopenia represents one of the hallmarks of all chronic diseases, including cancer, and was already investigated as a prognostic marker in the *pre-immunotherapy era*. Sarcopenia can be evaluated using cross-sectional image analysis of CT-scans, at the level of the third lumbar vertebra (L3), to estimate the skeletal muscle index (SMI), a surrogate of skeletal muscle mass, and to evaluate the skeletal muscle density (SMD). We performed a retrospective analysis of consecutive advanced cancer patient treated with PD-1/PD-L1 checkpoint inhibitors. Baseline SMI and SMD were evaluated and optimal cut-offs for survival, according to sex and BMI (+/−25) were computed. The evaluated clinical outcomes were: objective response rate (ORR), immune-related adverse events (irAEs), progression free survival (PFS) and overall survival (OS). From April 2015 to April 2019, 100 consecutive advanced cancer patients were evaluated. 50 (50%) patients had a baseline low SMI, while 51 (51%) had a baseline low SMD according to the established cut offs. We found a significant association between SMI and ECOG-PS (p = 0.0324), while no correlations were found regarding SMD and baseline clinical factors. The median follow-up was 20.3 months. Patients with low SMI had a significantly shorter PFS (HR = 1.66 [95% CI: 1.05–2.61]; p = 0.0291) at univariate analysis, but not at the multivariate analysis. They also had a significantly shorter OS (HR = 2.19 [95% CI: 1.31–3.64]; p = 0.0026). The multivariate analysis confirmed baseline SMI as an independent predictor for OS (HR = 2.19 [1.31–3.67]; p = 0.0027). We did not find significant relationships between baseline SMD and clinical outcomes, nor between ORR, irAEs and baseline SMI (data not shown). Low SMI is associated with shortened survival in advanced cancer patients treated with PD1/PDL1 checkpoint inhibitors. However, the lack of an association between SMI and clinical response suggests that sarcopenia may be generally prognostic in this setting rather than specifically predictive of response to immunotherapy.

## Introduction

Sarcopenia is the condition of loss of muscle mass, with decreased muscle power, and it is one of the hallmarks of cancer, which negatively affects the most of clinical outcomes such as toxicities and survival^1^. The interactions between malnutrition, cachexia and inflammation have been widely investigated and are still matter of debate^2^. In cancer patients, the skeletal muscle index (SMI) is widely used as surrogate of the body muscle mass (and sarcopenia), and is often evaluated through cross‐sectional image analysis from CT (computed tomography) scans^1^. The SMI, together with the skeletal muscle radiodensity (SMD), which is used to quantify muscle degradation and myosteatosis, have already revealed to be prognostic and predictive parameters in cancer patients^1,3^.

Considering that a negative influence of body composition alterations and sarcopenia on declining immunity has already been assumed^4^, it is becoming clearer that after the advent of immune checkpoint inhibitors (ICIs), the body composition evaluation could regain importance. For example, skeletal muscle cells might modulate immune response in health and disease (autoimmune diseases particularly)^5^, interacting with immune cells like non-professional antigen presenting cells (APCs), and expressing major histocompatibility complexes I and II^5^. It is been already reported that sarcopenic melanoma patients are more likely to experience immune related adverse events (irAEs)^6,7^. Recently, skeletal muscle mass has been included in prognostic score, which independently predicts survival in patients treated with anti PD-1/PD-L1 (programmed deadth-1/programmed death-ligand 1) agents^8^. In a preliminary report, we found that sarcopenic non-small cell lung cancer (NSCLC) patients receiving nivolumab had shorter progression free survival (PFS) and overall survival (OS)^9^. Moreover, other two retrospective studies found a significant association between sarcopenia, shorter PFS and worse objective response rate (ORR)^10,11^.

Here we present the results of a multicenter retrospective study of advanced cancer patients treated with PD-1/PD-L1 checkpoint inhibitors with a baseline evaluation of SMI and SMD.

## Materials and Methods

### Anthropometric measurements and image analysis

Patients were eligible if they had confirmed diagnosis of measurable advanced cancer, with available imaging assessment (CT or Positron Emission Tomography-Computed Tomography), performed before starting the immunotherapy (no more than three months earlier). Muscle mass was measured within CT images. Axial images of abdomen were analyzed in a workstation using OSIRIX-Lite software V5.0 (Pixmeo, Sarl, Switzerland) by a trained observer (PP), blinded to patient outcomes who reviewed all images. CT scan included acquisition from the lower chest areas to the pelvic floor. Slice thickness 3 mm/spacing 0.3 mm images were preferred to volumetric images (0,5 mm/0 mm) due to the intrinsic post-processing software limitations. In order to avoid the post-contrast muscle enhancement, which significantly increase after contrast media injection (arterial or early portal-venous phases)^12^, basal, or arterial phases at most, were used.

The third lumbar vertebra (L3), with both transverse processes visible, was chosen as the standard landmark. Skeletal muscle was quantified based on Hounsfield Unit (HU) thresholds (−29 to +150), than the SMI (cm^2^/m^2^) was computed dividing the total cross-sectional skeletal muscle area (TMA - cm^2^) at the level of L3, by squared height, because the TMA is linearly related to whole body muscle mass. The TMA was computed for each patient with semi-automated specific tissue demarcation of the muscles in the L3 region (psoas, paraspinal, and abdominal wall muscles, excluding visceral organs). If other structures apart those constituting TMA were automatically marked, they were eliminated by manual corrections. SMD was assessed as the mean radiodensity (HU) of the entire cross sectional muscle area at L3.

Given to the emerging association between BMI, patients sex and clinical outcomes of cancer patients receiving immunotherapy^13,14^, we did not used the already available sex-specific, BMI-incorporated, cut offs values for SMI and muscle attenuation^15^, which were established before the advent of immune checkpoint inhibitors. On the other hand, several correlations between sex^16^, BMI^3,17^ and skeletal muscle are already known. Moreover, SMI and BMI are inevitably related, because they are both computed with the squared height as denominator. Therefore, we computed new cut offs in the study population, according to the following subgroups: overweight (BMI > 25) males, non-overweight (BMI ≤ 25) males, overweight females, and non-overweight females. We then categorized patients in low SMI (which stands for sarcopenic) and non-low SMI, and low SMD and non-low SMD.

### Study design

This is a retrospective, multicenter, observational analysis of advanced cancer patients treated with anti-PD-1/PD-L1 agents in clinical practice, regardless of treatment line. Patients were treated according to the tumor type indication with pembrolizumab, nivolumab or atezolizumab and others PD-1/PD-L1 agents with standard doses and schedules. The aim of this study was to evaluate the correlations between baseline SMI and SMD and the following clinical outcomes: ORR, irAEs of any grade, PFS and OS. ORR was defined as the portion of patients experiencing an objective response (complete response, CR, or partial response, PR) as best response, measured by RECIST 1.1^18^. PFS was defined as the time elapsed between treatment initiation and disease progression or death from any cause; OS as the length of time between the beginning of treatment and death from any cause. Median PFS and median OS were evaluated using the Kaplan-Meier method, which was used also to estimate the time of treatment duration among subgroups. Median period of follow-up was calculated according to the reverse Kaplan-Meier method. Immune-related AEs were defined as those AEs having an immunological basis. They were graded according to the National Cancer Institute Common Toxicity Criteria for Adverse Events (CTCAE; version 4.0) and cumulatively reported as crude incidence. Chi-square was used to correlate ORR and the incidences of irAEs with baseline SMI and SMD. To find the optimal cut offs, Cox proportional hazard regression was used to compute the predicted probabilities for OS of both SMI and SMD (used as continuous variables) in the above mentioned pre-specified subgroups. Than the ROC curve with the area under the curve (AUC) for each variable were calculated, and the optimal cut offs for survival were determined using Youden’s J statistic.

The following clinical factors were evaluated: BMI (obese, overweight, normal weight, underweight), primary tumor (NSCLC, melanoma, kidney and others), age (<70 *vs* ≥70 years old)^19–22^, sex (male *vs* female), Eastern Cooperative Oncology Group Performance Status (ECOG-PS) (0–1 *vs* ≥2), burden of disease (number of metastatic sites ≤2 *vs* >2) and treatment line (first *vs* non-first). In order to properly weighing the impact on clinical outcomes and to find appropriate covariates, the correlations between SMI and SMD (according the study cut offs) and baseline clinical factors (primary tumor, age, ECOG-PS, burden of disease and treatment line) were evaluated with the chi-square test. Cox regression was used for univariate and multivariate analysis of PFS and OS. Sex, BMI and baseline clinical factors which were related to SMI and SMD were not used in the multivariate analyses^23^. In order to further evaluate the possible different role of body composition alterations in different tumor types, we performed the univariate efficacy analysis in NSCLC and melanoma patients cohorts separately. Data cut-off period was June 2019. All statistical analyses were performed using MedCalc Statistical Software version 19.0.4 (MedCalc Software bvba, Ostend, Belgium; https://www.medcalc.org; 2019).

### Ethics approval and consent to participate

All patients provided written, informed consent to treatment with immunotherapy. All patients alive at the time of data collection provided an informed consent for the present retrospective analysis. The procedures followed were in accordance with the precepts of Good Clinical Practice, and the declaration of Helsinki. Being a retrospective update of data previously collected, approval by institutional review boards was not required, although a notification was sent (normative ref. Gazzetta Ufficiale della Repubblica Italiana n. 76 of 31–3–2008) to the local responsible committee on human experimentation (University of L’Aquila, Internal Review Board protocol number 32865, approved on July 24th, 2018).

## Results

### Patients’ features

From April 2015 to April 2019, 100 consecutive advanced cancer patients, receiving anti-PD-1/PD-L1 checkpoint inhibitors at the oncology departments of St Salvatore Hospital in L’Aquila and SS Annunziata Hospital in Chieti, were eligible for the imaging analysis.

Patients’ characteristics are summarized in Table 1. 50 (50%) patients had a baseline low SMI based on optimal cut-offs, while 51 (51%) had a baseline low SMD according to previously established cut offs. We found a significant association between SMI and ECOG-PS (p = 0.0324), while no correlations were found regarding SMD and baseline clinical factors. The computed optimal cut offs for survival are listed in Table 2.

**Table 1:** Patients characteristics according to subgroups. P-values were obtain with the Chi-square test.

Patients - n° (%)	Overall	Low SMI	NON-Low SMI	p-value	Low SMD	NON-Low SMD	p-value
	100	50	50		51	49	
Age, years							
Range	25–88	27–88	36–86	—	40–86	27–88	—
Median	66	71	70		71	70	
Sex							
Male	67 (67)	31 (62)	36 (72)	—	40 (78.4)	27 (55.1)	—
Female	33 (33)	19 (38)	14 (28)		11 (21.6)	22 (44.9)	
Age (<70 ≥)							
Non Elderly	43 (43)	19 (38)	24 (48)	0.315	21 (41.2)	22 (44.9)	0.7085
Elderly	57 (57)	31 (62)	26 (53)		30 (58.8)	27 (55.1)	
ECOG PS							
0–1	59 (59)	22 (44)	37 (74)	0.0324	28 (54.9)	31 (63.3)	0.3977
≥2	41 (41)	28 (56)	13 (26)		23 (45.1)	18 (36.7)	
Primary tumor							
NSCLC	46 (46)	22 (44)	24 (48)	0.5471	27 (52.9)	19 (38.8)	0.5549
Melanoma	27 (27)	13 (26)	14 (28)		12 (23.5)	15 (30.6)	
Renal Cell Carcinoma	15 (46)	10 (20)	5 (10)		7 (13.7)	8 (16.3)	
Others	12 (12)	5 (10)	7 (14)		5 (9.8)	7 (14.3)	
No. of metastatic sites							
≤2	55 (55)	26 (52)	29 (58)	0.5485	27 (52.9)	28 (57.1)	0.6744
>2	45 (45)	24 (48)	21 (42)		24 (47.1)	21 (42.9)	
Treatment Line							
First	30 (30)	12 (24)	18 (36)	0.1927	14 (27.5)	16 (32.7)	0.5723
Non-first	70 (70)	38 (76)	32 (64)		37 (72.5)	33 (67.3)	
Type of Immunotherapy							
Anti-PD-1	91 (91)	48 (96)	43 (86)	—	46 (90.2)	45 (91.8)	—
Anti-PD-L1	9 (9)	2 (4)	7 (14)		5 (9.8)	4 (8.2)	
BMI (kg/m^2^)							
Median (range)	25 (17.3–45.2)	24 (16.4–39)	27 (17.1–45)	—	27 (18–45)	24 (16.4–34)	—
Underweight (BMI ≤ 18.5), n°(%)	5 (5)	3 (6)	2 (4)		1 (2)	4 (8.2)	
Normal weight (BMI 18.5 < BMI ≤ 24.9), n°(%)	41 (41)	24 (48)	17 (34)		11 (21.6)	30 (61.2)	
Overweight (25 < BMI ≤ 29.9), n°(%)	33 (33)	17 (34)	16 (32)		23 (45.1)	10 (20.4)	
Obese (BMI ≥ 30), n° (%)	21 (21)	6 (12)	15 (30)		16 (31.3)	5 (10.2)	
SMI (cm^2^/m^2^)							
Median	48.2	42.7	56.5	—	49.5	45	—
(range)	(28.2–95.2)	(28.2–56.8)	(36.9–95.9)		(28.2–85.8)	(32.8–95.9)	
SMD (HU)							
Median	30.9	29.9	31.6	—	23.6	37.9	—
(range)	(2.3–54.6)	(5.9–53.1)	(2.3–54.6)		(2.3–36.2)	(24.2–54.6)

**Table 2:** SMI and SMD computed optimal cut-offs according to sex and BMI in the study population.

BMI category	SMI (cm^2^/m^2^)		SMD (HU)	
	Male	Female	Male	Female
Overweight (≥25)	>50.2	>59.6	>35.6	>37.4
Non-overweight (<25)	>48.4	>36.9	>24.2	>27.9

### Clinical outcomes analysis (overall study population)

**Figure 1:**
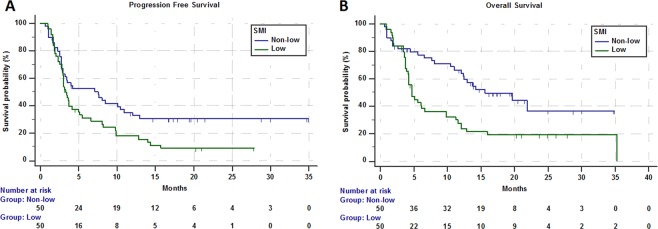
Kaplan-Meier survival curves according to SMI category. (**A**) Progression Free Survival. (**B**) Overall Survival.

**Figure 2:**
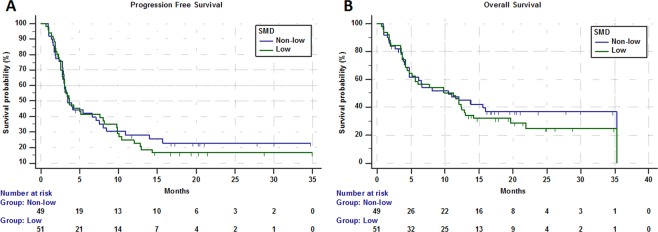
Kaplan-Meier survival curves according to SMD category. (**A**) Progression Free Survival. (**B**) Overall Survival.

In the study population ORR was 23.2% (95% CI: 14.5–35.1; 22 PR out of 95 evaluable patients). Table 3 summarized the subgroup analysis of ORR. At the data cut-off 21 patients were still on treatment. Median time of treatment duration in the overall study population was 3.4 months (95% CI: 2.9–5), while in low and non-low SMI subgroups was 3.1 months (95% CI: 2.8–4.1) and 3.7 months (95% CI: 2.8–10.4), respectively. Among low ad non low-SMD subgroups the time of treatment duration was 3.6 months (95% CI: 2.8–6.1) and 3.2 months (95% CI: 2.8–5.7), respectively. The median follow-up was 20.3 months; in the study population median PFS and median OS were 3.7 months (95% CI: 3.1–7.1; 77 events) and 10.4 months (95% CI: 5.6–12.9; 34 censored patients). Median PFS and median OS of patients with low SMI were 3.3 months (95% CI: 2.8–5; 44 events) and 4.7 months (95% CI: 4.1–6.6; 9 censored) respectively (Fig. 1). Median PFS and OS of patients with non-low SMI were 7.5 months (95% CI: 2.9–10.9; 33 events) and 15.6 months (95% CI: 12–21.9; 25 censored), respectively (Fig. 1). Median PFS and OS of patients with low SMD were 3.7 months (95% CI: 2.8–8.1; 41 events) and 11.2 months (95% CI: 4.7–12.9; 14 censored) respectively (Fig. 2). Median PFS and OS of patients with non-low SMD were 3.5 months (95% CI: 2.9–7.5; 36 events) and 10.4 months (95% CI: 4.7–35.3; 20 censored), respectively (Fig. 2).

**Table 3:** ORR analysis according to SMI and SMD categories.

Variable	Response/Ratio	ORR (95% CI)	*p - value*
**Overall**	22/95	23.2 (14.5–35.1)	—
SMI			
Low	11/48	22.9 (11.4–41.0)	*0.9553*
Non-low	11/47	23.4 (11.6–41.8)	
SMD			
Low	8/49	16.3 (7.1–32.1)	*0.1051*
Non-low	14/46	30.4 (16.6–51.1)

Table 4 summarized univariate and multivariate analyses of PFS. Patients with low SMI had a significantly shorter PFS (HR = 1.66 [95% CI: 1.05–2.61]; p = 0.0291) at univariate analysis, but not at the multivariate analysis; baseline SMD was not related to PFS. Table 5 summarized univariate and multivariate analyses of OS. Patients with low SMI had a significantly shorter OS at univariate analysis (HR = 2.19 [95% CI: 1.31–3.64]; p = 0.0026); the multivariate analysis confirmed baseline SMI as an independent predictor for OS (HR = 2.19 [1.31–3.67]; p = 0.0027). Baseline SMD was not significantly related to OS.

**Table 4:** Univariate and multivariate analysis of Progression Free Survival.

Variable (comparator)	Progression Free Survival			
	Univariate Analysis		Multivariate Analysis (SMI)	
	HR (95% CI)	*p-value*	HR (95% CI)	*p-value*
**SMI**				
low vs non-low	1.66 (1.05–2.61)	*0.0291*	1.48 (0.93–2.38)	*0.0968*
**SMD**				
low vs non-low	1.09 (0.69–1.71)	*0.7023*	—	—
**Age at diagnosis**				
Elderly vs non-elderly	1.13 (0.72–1.79)	*0.5757*	—	—
**ECOG-PS**				
**≥**2 vs 0–1	3.73 (2.29–6.07)	<*0.0001*	—	—
**Primary Tumor**				
(NSCLC)				
Melanoma	0.73 (0.41–1.27)	*0.2723*	0.96 (0.51–1.83)	*0.9168*
Renal cell carcinoma	0.96 (0.51–1.81)	*0.9049*	0.49 (0.25–0.96)	*0.0388*
Others	0.74 (0.35–1.55)	*0.4320*	0.75 (0.35–1.61)	*0.4677*
**No. of metastatic site**				
**>**2 vs ≤2	2.71 (1.68–4.38)	<*0.0001*	3.25 (1.93–5.47)	<*0.0001*
**Treatment line**				
Non-first vs First	2.22 (1.29–3.83)	*0.0038*	2.83 (1.46–5.47)	*0.0020*

**Table 5:** Univariate and multivariate analysis of Overall Survival.

Variable (comparator)	Overall Survival			
	Univariate Analysis		Multivariate Analysis (SMI)	
	HR (95% CI)	*p - value*	HR (95% CI)	*p - value*
**SMI**				
low vs non-low	2.19 (1.31–3.64)	*0.0026*	2.19 (1.31–3.67)	*0.0027*
**SMD**				
low vs non-low	1.15 (0.71–1.87)	*0.5618*	—	—
**Age at diagnosis**				
Elderly vs non-elderly	0.95 (0.58–1.56)	*0.8443*	—	—
**ECOG-PS**				
**≥**2 vs 0–1	7.15 (4.12–12.41)	<*0.0001*	—	—
**Primary Tumor**				
(NSCLC)				
Melanoma	1.03 (0.57–1.86)	*0.9023*	1.01 (0.56–1.83)	*0.9588*
Renal cell carcinoma	1.06 (0.51–2.16)	*0.8791*	0.85 (0.41–1.76)	*0.6636*
Others	0.75 (0.31–1.81)	*0.5263*	1.01 (0.40–2.51)	*0.9904*
**No. of metastatic site**				
**>**2 vs ≤2	2.07 (1.26–3.43)	*0.0042*	2.06 (1.22–3.51)	*0.0073*
**Treatment line**				
Non-first vs First	1.65 (0.91–2.99)	*0.0956*	—	—

Twenty-five (25%) patients experienced irAEs of any grade in the overall population. Among patients with low and non-low SMI, 11 (22%) and 14 (28%) experienced irAEs of any grade, respectively (p = 0.4906). Among patients with low and non-low SMD, 10 (19.6%) and 15 (30.6%) experienced irAEs of any grade, respectively (p = 0.2062).

### Efficacy analysis of NSCLC and melanoma patients cohorts

No significant differences were observed regarding ORR according to the SMI nor regarding ORR, PFS and OS according to the SMD, in both the NSCLC and melanoma cohorts (data not reported).

Median PFS and median OS among melanoma patients were 5.4 months (95% CI: 3.4–8; 19 events) and 8.1 months (95% CI: 4.7–12.9; 9 censored), respectively. Median PFS of patients with low SMI and non-low SMI was 3.6 months (95% CI: 2.5–5.4; 11 events) and 8.0 months (95% CI: 2.5–12.9; 8 events), respectively. The difference was not statistically significant (HR = 2.5 [95% CI: 0.95–6.36], p = 0.0626). Median OS of patients with low SMI and non-low SMI was 4.7 months (95% CI: 3.5–12; 2 censored) and 13.8 months (95% CI: 5.6–13.8; 7 censored), respectively. The difference was statistically significant (HR = 3.11 [95% CI: 1.16–8.33], p = 0.0237).

Median PFS and median OS among NSCLC patients were 3.0 months (95% CI: 2.8–6.6; 36 events) and 11.2 months (95CI: 4.7–19.7; 14 censored), respectively. Median PFS of low and non-low SMI patients was 3.0 months (95% CI: 1.8–5.1; 20 events) and 3.9 months (95% CI: 2.5–11.9; 16 events), respectively. The difference was not statistically significant (HR = 1.55 [95% CI; 0.79–3.02], p = 0.1930). Median OS of low and non-low SMI patients was 4.7 months (95% CI: 1.8–11.5; 3 censored) and 15.6 months (95% CI: 7.7–21.9; 11 censored), respectively. The difference was not statistically significant (HR = 1.81 [95% CI: 0.87–3.76], p = 0.1098).

## Discussion

In our population, patients with low SMI had a significantly shorter PFS only at the univariate analysis, while had a significantly shorter OS at both univariate and multivariate analyses. On the other hand we did not find significant relationships between ORR, irAEs and baseline SMI, nor between baseline SMD and any of the measured clinical outcomes. The absence of significant correlation with ORR suggests that sarcopenia does not have a predictive value to immunotherapy, while has prognostic role overall, which persists even during PD-1/PD-L1 inhibitors.

The SMD can be used to provide a qualitative, rather than quantitative (as the SMI), estimation of skeletal muscle composition; it asses distribution of adipose tissue (myosteatosis), muscle atrophy/wasting caused by or associated with inactivity, denervation, and chronic diseases^24^. The role of SMD as a predictive and prognostic parameter still remains uncertain in cancer patients, compared to other chronic disorders, but surely retain its importance in identifying more frail patients, with body composition alterations^1,3^.

Interestingly, we found a significant difference in median OS according to the SMI only in the melanoma patients cohort, while not among NSCLC patients. The small sample size of the two cohorts could have affect the results, however the melanoma cohort was smaller than the NSCLC cohort. In our opinion, these difference might be related not so much to the intrinsic disease characteristics, but to the different efficacy profiles of PD-1/PD-L1 inhibitors and to the different confidence intervals among the two cohorts.

The decreasing in muscle mass and muscle deterioration are features of many chronic diseases. Then, we must not be surprised by the significant correlation between poorer PS and low-SMI. Even if not significant, a higher percentage of poorer PS patients was also found among whose with low SMD. Indeed, PS is a measure of patients well-being and activities of daily life, which of course are related to skeletal muscle and muscle power. Therefore, we must recognize the prevalent role of PS, which is related to SMI. Looking at the Table 4, we can noticed that the ECOG-PS is the factor with the highest hazard ratio for OS at the univariate analysis (7.15), so we can assume that sarcopenic patients had a shorter OS, because they basically had poorer clinical conditions.

Shiroyama and colleagues evaluated a little cohort (42) of NSCLC patients treated with PD-1 checkpoint inhibitors, without finding a significant association between sarcopenia and ECOG-PS, probably due to the small sample size^10^. Despite that, when they adjusted the multivariate analysis of PFS by sex and PS, sarcopenia did not retain the statistical significance^10^. Similarly, Nishioka and colleagues evaluated the change in muscle mass over time in 38 NSCLC patients receiving PD-1 Inhibitors^11^. Even if not significant, they found a higher change rate (decrease in muscle mass) among patients with poorer PS (p = 0.056)^11^.

Two studies have already reported a higher incidence of adverse events in sarcopenic melanoma patients treated with PD-1 inhibitors^6^ and ipilimumab^7^, but we did not find any correlations between irAEs and SMI, nor between irAEs and SMD. Even if sarcopenia has been associated with a greater incidence of chemotherapy toxicity^1^, things might be different with the irAEs. Recent findings suggest that being a pharmacodynamic resultance, the occurence of irAEs could be considered a biomarker of immunotherapy efficacy across different tumor tipes^25–28^. From this perspective, patients who are likely to benefit more from ICIs treatments, should be the same who are more likely to experience irAEs, so we might speculate that with ICIs, sarcopenic patients should experience less irAEs compared to non-sarcopenic patients.

It is known that body composition and sex affect the immune system^16,17^, and several studies have already investigated the complex inter-relationships between BMI, sex and clinical outcomes with ICIs^13,14,29^. The aim of our study was to assess whether (and how) the skeletal muscle (sarcopenia and muscle degradation) affected immunotherapy clinical outcomes, not the role of BMI. Therefore, since in a previous study with a similar population we revealed that a BMI ≥ 25 has a positive predictive and prognostic role during immunotherapy^13^, we used BMI +/− 25 as a landmark to categorize patients while computing the SMI and SMD optimal cut-offs. On the other hand, the median age of our study population was 66 years and 57% of the patients were elderly, and we must recognize that some authors have already speculated about the age-dependent relationship between BMI and mortality overall (non-cancer patients) in both sex^30,31^.

The prognostic weight of sarcopenia seems to particularly affect obese patients^32^; this hypothesis might not appear aligned to the recent evidences suggesting a positive predictive and prognostic role of a high BMI during ICI therapy^13,33,34^. Recently, an interesting study have tried to shed a light on the complex inter-relations between sex, BMI and sarcopenia, in melanoma patients treated with ICIs (using serum creatinine as a surrogate of muscle mass)^35^. The authors intriguingly found that the best clinical outcome is achieved in overweight/class I obese patients (BMI 25–35), particularly among males, who had higher serum creatinine levels (which stands for a good muscle mass)^35^. BMI and muscle mass seem to have a direct proportionality (the higher is the BMI, the higher the SMI), vice versa their effect on immunotherapy clinical outcomes appears opposite (high BMI has a positive predictive and prognostic role, while sarcopenia has a negative prognostic role), which overlaps in a specific subset of patients (overweight non sarcopenic). Considering the easy availability of baseline CT scans for each cancer patient, and the clinical utility of estimate body composition alterations (malnourished/frail patients), SMI and SMD might be evaluated in clinical practice. However, softwares and acquisition protocols must be validate in dedicated trials before their rountinary use could be allowes.

Our study has several limitations, such as the retrospective design, which exposes us to the risk of selection bias, and the sample size, which might be small for a proper evaluation of the prognostic weight of sarcopenia. We must recognize also the lack of other adiposity metrics, such as the waist circumference, the waist-to-height ratio, and the body fat percentage. Moreover, the CT imaging analysis was limited by the data availability; indeed, the acquisition protocol was planned according to the presence of previous examination.

## Conclusion

Our finding of a significant shorter OS for low-SMI patients treated with PD-1/PD-L1 checkpoint inhibitors, suggests that sarcopenia might have a prognostic role, rather than predictive. However, to properly weighing our results, we must consider the significant association between poorer PS and low-SMI. Without making conclusive considerations, we can assume that after the advent of ICIs, we should give back further relevance to baseline nutritional (and body composition) assessment of every patient.

## Data Availability

The datasets used during the present study are available from the corresponding author upon reasonable request.

## References

[1] Bozzetti F. (2017). Forcing the vicious circle: sarcopenia increases toxicity, decreases response to chemotherapy and worsens with chemotherapy. Annals of Oncology.

[2] Laviano, A., Koverech, A. & Mari, A. Cachexia: clinical features when inflammation drives malnutrition. *Proc Nutr Soc*. **74**(4), 348–54 (2015). 10.1017/S0029665115000117 25809872

[3] Cortellini Alessio, Palumbo Pierpaolo, Porzio Giampiero, Verna Lucilla, Giordano Aldo V., Masciocchi Carlo, Parisi Alessandro, Cannita Katia, Ficorella Corrado, Bozzetti Federico (2018). Single-institution study of correlations between skeletal muscle mass, its density, and clinical outcomes in non-small cell lung cancer patients treated with first-line chemotherapy. Thoracic Cancer.

[4] Lutz, C. T. & Quinn, L. S. Sarcopenia, obesity, and natural killer cell immune senescence in aging: altered cytokine levels as a common mechanism. *Aging (Albany NY)*. **4**(8), 535–46 (2012). 10.18632/aging.100482 PMC3461341 22935594

[5] Afzali Ali Maisam, Müntefering Thomas, Wiendl Heinz, Meuth Sven G., Ruck Tobias (2018). Skeletal muscle cells actively shape (auto)immune responses. Autoimmunity Reviews.

[6] Heidelberger Valentine, Goldwasser François, Kramkimel Nora, Jouinot Anne, Huillard Olivier, Boudou-Rouquette Pascaline, Chanal Johan, Arrondeau Jennifer, Franck Nathalie, Alexandre Jérôme, Blanchet Benoît, Leroy Karen, Avril Marie-Françoise, Dupin Nicolas, Aractingi Sélim (2017). Sarcopenic overweight is associated with early acute limiting toxicity of anti-PD1 checkpoint inhibitors in melanoma patients. Investigational New Drugs.

[7] Daly Louise E, Power Derek G, O'Reilly Áine, Donnellan Paul, Cushen Samantha J, O'Sullivan Kathleen, Twomey Maria, Woodlock David P, Redmond Henry P, Ryan Aoife M (2017). The impact of body composition parameters on ipilimumab toxicity and survival in patients with metastatic melanoma. British Journal of Cancer.

[8] Dercle L (2016). Rapid and objective CT scan prognostic scoring identifies metastatic patients with long‐term clinical benefit on anti‐PD‐1/‐L1 therapy. Eur. J. Cancer.

[9] Cortellini Alessio, Verna Lucilla, Porzio Giampiero, Bozzetti Federico, Palumbo Pierpaolo, Masciocchi Carlo, Cannita Katia, Parisi Alessandro, Brocco Davide, Tinari Nicola, Ficorella Corrado (2019). Predictive value of skeletal muscle mass for immunotherapy with nivolumab in non-small cell lung cancer patients: A “hypothesis-generator” preliminary report. Thoracic Cancer.

[10] Shiroyama T (2019). Impact of sarcopenia in patients with advanced non–small cell lung cancer treated with PD-1 inhibitors: A preliminary retrospective study. Sci. Rep..

[11] Nishioka Naoya, Uchino Junji, Hirai Soichi, Katayama Yuki, Yoshimura Akihiro, Okura Naoko, Tanimura Keiko, Harita Sachi, Imabayashi Tatsuya, Chihara Yusuke, Tamiya Nobuyo, Kaneko Yoshiko, Yamada Tadaaki, Takayama Koichi (2019). Association of Sarcopenia with and Efficacy of Anti-PD-1/PD-L1 Therapy in Non-Small-Cell Lung Cancer. Journal of Clinical Medicine.

[12] Morsbach Fabian, Zhang Yi-Hua, Martin Lena, Lindqvist Catarina, Brismar Torkel (2019). Body composition evaluation with computed tomography: Contrast media and slice thickness cause methodological errors. Nutrition.

[13] Cortellini, A. *et al*. A multicenter study of body mass index in cancer patients treated with anti-PD-1/PD-L1 immune checkpoint inhibitors: when overweight becomes favorable. *J Immunother Cancer*. **7**(1), 57, 10.1186/s40425-019-0527-y (2019). 10.1186/s40425-019-0527-y PMC6391761 30813970

[14] Conforti Fabio, Pala Laura, Bagnardi Vincenzo, De Pas Tommaso, Martinetti Marco, Viale Giuseppe, Gelber Richard D, Goldhirsch Aron (2018). Cancer immunotherapy efficacy and patients' sex: a systematic review and meta-analysis. The Lancet Oncology.

[15] Martin Lisa, Birdsell Laura, MacDonald Neil, Reiman Tony, Clandinin M. Thomas, McCargar Linda J., Murphy Rachel, Ghosh Sunita, Sawyer Michael B., Baracos Vickie E. (2013). Cancer Cachexia in the Age of Obesity: Skeletal Muscle Depletion Is a Powerful Prognostic Factor, Independent of Body Mass Index. Journal of Clinical Oncology.

[16] Anderson LJ, Liu H, Garcia JM (2017). Sex Differences in Muscle Wasting. Adv. Exp. Med. Biol..

[17] Abramowitz MK (2018). Muscle mass, BMI, and mortality among adults in the United States: A population-based cohort study. PLoS One..

[18] Eisenhauer, E. A. *et al*. New response evaluation criteria in solid tumours: revised RECIST guideline (version 1.1). *Eur J Cancer*. (2009). 10.1016/j.ejca.2008.10.026 19097774

[19] Minana, B. *et al*. Bladder cancer in Spain 2011: population-based study. *J Urol* **191**(2), 323–8 (2014). 10.1016/j.juro.2013.08.049 23994371

[20] Ciocan Dragos, Barbe Coralie, Aubin Francois, Granel-Brocard Florence, Lipsker Dan, Velten Michel, Dalac Sophie, Truchetet François, Michel Catherine, Mitschler Audrey, Arnoult Gwendoline, Buemi Antoine, Dalle Stéphane, Bernard Philippe, Woronoff Anne-Sophie, Grange Florent (2013). Distinctive Features of Melanoma and Its Management in Elderly Patients. JAMA Dermatology.

[21] Gridelli Cesare, Balducci Lodovico, Ciardiello Fortunato, Di Maio Massimo, Felip Enriqueta, Langer Corey, Lilenbaum Rogerio C., Perrone Francesco, Senan Suresh, de Marinis Filippo (2015). Treatment of Elderly Patients With Non–Small-Cell Lung Cancer: Results of an International Expert Panel Meeting of the Italian Association of Thoracic Oncology. Clinical Lung Cancer.

[22] Azawi NH (2016). Trends in Kidney cancer among the elderly in Denmark, 1980–2012. Acta Oncol..

[23] Bonate PL (2017). Effect of correlation on covariate selection in linear and nonlinear mixed effect models. Pharmaceut. Statist..

[24] Engelke Klaus, Museyko Oleg, Wang Ling, Laredo Jean-Denis (2018). Quantitative analysis of skeletal muscle by computed tomography imaging—State of the art. Journal of Orthopaedic Translation.

[25] Freeman-Keller M., Kim Y., Cronin H., Richards A., Gibney G., Weber J. S. (2015). Nivolumab in Resected and Unresectable Metastatic Melanoma: Characteristics of Immune-Related Adverse Events and Association with Outcomes. Clinical Cancer Research.

[26] Haratani Koji, Hayashi Hidetoshi, Chiba Yasutaka, Kudo Keita, Yonesaka Kimio, Kato Ryoji, Kaneda Hiroyasu, Hasegawa Yoshikazu, Tanaka Kaoru, Takeda Masayuki, Nakagawa Kazuhiko (2018). Association of Immune-Related Adverse Events With Nivolumab Efficacy in Non–Small-Cell Lung Cancer. JAMA Oncology.

[27] Cortellini Alessio, Chiari Rita, Ricciuti Biagio, Metro Giulio, Perrone Fabiana, Tiseo Marcello, Bersanelli Melissa, Bordi Paola, Santini Daniele, Giusti Raffaele, Grassadonia Antonino, Di Marino Pietro, Tinari Nicola, De Tursi Michele, Zoratto Federica, Veltri Enzo, Malorgio Francesco, Garufi Carlo, Russano Marco, Anesi Cecilia, Zeppola Tea, Filetti Marco, Marchetti Paolo, Berardi Rossana, Rinaldi Silvia, Tudini Marianna, Silva Rosa Rita, Pireddu Annagrazia, Atzori Francesco, Iacono Daniela, Migliorino Maria Rita, Porzio Giampiero, Cannita Katia, Ficorella Corrado, Buti Sebastiano (2019). Correlations Between the Immune-related Adverse Events Spectrum and Efficacy of Anti-PD1 Immunotherapy in NSCLC Patients. Clinical Lung Cancer.

[28] Cortellini Alessio, Buti Sebastiano, Agostinelli Veronica, Bersanelli Melissa (2019). A systematic review on the emerging association between the occurrence of immune-related adverse events and clinical outcomes with checkpoint inhibitors in advanced cancer patients. Seminars in Oncology.

[29] Warner, A. B & McQuade, J. L. Modifiable Host Factors in Melanoma: Emerging Evidence for Obesity, Diet, Exercise, and the Microbiome. *Curr Oncol Rep*. **21**(8), 72, 10.1007/s11912-019-0814-2 (2019). 10.1007/s11912-019-0814-2 PMC7472428 31263961

[30] Ng TP (2017). Age-dependent relationships between body mass index and mortality: Singapore longitudinal ageing study. PLoS One..

[31] Kvamme JM (2012). Body mass index and mortality in elderly men and women: the Tromso and HUNT studies. J. Epidemiol. Community Health..

[32] Gonzalez MC (2014). Obesity paradox in cancer: New insights provided by body composition. Am. J. Clin. Nutr..

[33] McQuade Jennifer L, Daniel Carrie R, Hess Kenneth R, Mak Carmen, Wang Daniel Y, Rai Rajat R, Park John J, Haydu Lauren E, Spencer Christine, Wongchenko Matthew, Lane Stephen, Lee Dung-Yang, Kaper Mathilde, McKean Meredith, Beckermann Kathryn E, Rubinstein Samuel M, Rooney Isabelle, Musib Luna, Budha Nageshwar, Hsu Jessie, Nowicki Theodore S, Avila Alexandre, Haas Tomas, Puligandla Maneka, Lee Sandra, Fang Shenying, Wargo Jennifer A, Gershenwald Jeffrey E, Lee Jeffrey E, Hwu Patrick, Chapman Paul B, Sosman Jeffrey A, Schadendorf Dirk, Grob Jean-Jacques, Flaherty Keith T, Walker Dana, Yan Yibing, McKenna Edward, Legos Jeffrey J, Carlino Matteo S, Ribas Antoni, Kirkwood John M, Long Georgina V, Johnson Douglas B, Menzies Alexander M, Davies Michael A (2018). Association of body-mass index and outcomes in patients with metastatic melanoma treated with targeted therapy, immunotherapy, or chemotherapy: a retrospective, multicohort analysis. The Lancet Oncology.

[34] Wang Ziming, Aguilar Ethan G., Luna Jesus I., Dunai Cordelia, Khuat Lam T., Le Catherine T., Mirsoian Annie, Minnar Christine M., Stoffel Kevin M., Sturgill Ian R., Grossenbacher Steven K., Withers Sita S., Rebhun Robert B., Hartigan-O’Connor Dennis J., Méndez-Lagares Gema, Tarantal Alice F., Isseroff R. Rivkah, Griffith Thomas S., Schalper Kurt A., Merleev Alexander, Saha Asim, Maverakis Emanual, Kelly Karen, Aljumaily Raid, Ibrahimi Sami, Mukherjee Sarbajit, Machiorlatti Michael, Vesely Sara K., Longo Dan L., Blazar Bruce R., Canter Robert J., Murphy William J., Monjazeb Arta M. (2018). Paradoxical effects of obesity on T cell function during tumor progression and PD-1 checkpoint blockade. Nature Medicine.

[35] Naik, G. S. *et al*. Complex inter-relationship of body mass index, gender and serum creatinine on survival: exploring the obesity paradox in melanoma patients treated with checkpoint inhibition. *J. Immunother Cancer*. **7**, 89, 10.1186/s40425-019-0512-5 (2019). 10.1186/s40425-019-0512-5 PMC6440018 30922394

